# What is the value of explicit priority setting for health interventions? A simulation study

**DOI:** 10.1007/s10729-022-09594-4

**Published:** 2022-05-28

**Authors:** Euan Barlow, Alec Morton, Saudamini Dabak, Sven Engels, Wanrudee Isaranuwatchai, Yot Teerawattananon, Kalipso Chalkidou

**Affiliations:** 1 Department of Management Science, Strathclyde Business School, University of Strathclyde, Glasgow, UK; 2 Health Intervention and Technology Assessment Program (HITAP), Ministry of Public Health, Nonthaburi, Thailand; 3 Institute of Health Policy, Management and Evaluation, University of Toronto, Toronto, Canada; 4 Saw Swee Hock School of Public Health, National University of Singapore, Singapore, Singapore; 5 iDSI, School of Public Health, Imperial College London, London, UK; 6 Center for Global Development, Washington, DC USA

**Keywords:** Priority setting institutions, Health technology assessment, Cost-effectiveness thresholding, Portfolio decision analysis, Simulation

## Abstract

Many countries seek to secure efficiency in health spending through establishing explicit priority setting institutions (PSIs). Since such institutions divert resources from frontline services which benefit patients directly, it is legitimate and reasonable to ask whether they are worth the money. We address this question by comparing, through simulation, the health benefits and costs from implementing two alternative funding approaches – one scenario in which an active PSI enables cost-effectiveness-threshold based funding decisions, and a counterfactual scenario where there is no PSI. We present indicative results for one dataset from the United Kingdom (published in 2015) and one from Malawi (published in 2018), which show that the threshold rule reliably resulted in decreased health system costs, improved health benefits, or both. Our model is implemented in Microsoft Excel and designed to be user-friendly, and both the model and a user guide are made publicly available, in order to enable others to parameterise the model based on the local setting. Although inevitably stylised, we believe that our modelling and results offer a valid perspective on the added value of explicit PSIs.

## Highlights

- Portfolio simulation of health intervention funding decisions provides a quantitative evaluation of the value delivered by health care priority setting institutions (PSIs)
- Funding criteria that prioritise interventions based on their cost-effectiveness are compared with counterfactual funding criteria
- A range of cost-effectiveness-based decision rules are investigated, aiming to provide practical insights to PSIs using alternative funding approaches
- Potential insights on operationalising a PSI include the expected effectiveness of different funding strategies and on how these are best implemented
- This modelling approach could be used by policy makers and governments to justify and inform the establishment of new PSIs and to evaluate the effectiveness of established PSIs

## Introduction

Modern medicine offers greater potential for alleviating human suffering and expanding the lifespan than ever before. In a world of limited resources, however, hard choices have to be made about what to fund [9]. In rich countries, population ageing means that there are increased demands on health systems, which leaves little headroom for financing medical innovation. For these countries, rising expectations about availability and quality of services and financial protection of patients exceed the resources flowing into the system by a considerable margin.

Many countries have responded to this challenge by establishing Priority Setting Institutions (PSIs) [7]. The philosophy behind these priority setting agencies is that decisions about priority setting should be made in a transparent and accountable way, drawing on best evidence and clearly articulating the underpinning values. As well as being in line with established wisdom about good practice in health system governance, such explicit priority setting has the advantage that it sends a clear signal to manufacturers about the value of particular product characteristics, enabling them to better steer their R&D portfolios [11].

Historically, explicit priority setting in this manner is a relatively new idea, seen in the context of the centuries-long history of health services. For much of history, health services were delivered largely through the market on an out-of-pocket basis, with public or philanthropic funds allocated largely on the basis of the urgency of the need, or the deservingness or poverty of the recipient. In the first few decades of the UK’s National Health Services, cost control was achieved largely through local budgets and capacity restrictions determined on the basis of precedent, with waiting lists being used effectively as a demand management tool.

Against this backdrop, the establishment of PSIs represents a huge step forward in the application of the tools of epidemiological and clinical science to the management of health systems in order to secure the efficient and equitable delivery of health services [28]. Nevertheless, running such agencies is not costless, and the contribution which the staff of such agencies make to the health system is less visible to the general public than that of staff on the frontline.

In this paper we present a novel methodology which can give valuable insights into the contribution of PSIs to the nation and its health system. The remainder of the paper is organised as follows: In the Background section we provide more detailed motivation for our approach and outline the questions which we will address; in the Methodology section we outline the structure of the simulation model and the datasets which we use to parameterise the model; in the Results section we show how, while our results do generally suggest that PSIs add significant value, the precise quantum of the gain is influenced by modelling assumptions and situational factors; and in the Discussion and Conclusion sections, we draw the implications of our work for the practice of priority setting, and suggest some directions forward.

## Background

### Assessing the contribution of priority setting agencies

Several studies (reviewed in more detail in a companion paper [19]) have assessed the contribution of priority setting agencies in various countries, including the UK, Canada, Australia, the United States and the Netherlands [1, 24]. Existing studies can be classified as deploying either one of two broad methodological approaches, or deploying a mixture of these: *modelling studies* use economic models developed within the priority setting process itself to quantify the scale of the financial and health impact from interventions which have received a positive recommendation from the priority setting agency; whereas *implementation studies* examine whether changes in clinical practice have followed such positive recommendations. Such studies are illuminating and leave no doubt about the important role which PSIs play in the health systems of the countries in question.

Nevertheless, a weakness of most (or all) existing work is that it does not address directly the question of the counterfactual, that is, what would have happened in the absence of a PSI. The value of a PSI can only be gauged by the extent to which it recommends the acceptance of interventions which would otherwise be rejected, or the rejection of interventions which would otherwise be accepted.

One way to study the impact of PSIs which addresses the issue of the counterfactual would be to take a case–control approach, identifying countries which have instituted some form of PSI and comparing them with others at a similar stage of development which have not. Indeed, the argument which many advocates of explicit priority setting make is to compare the overall system costs in the US market with system costs in the likes of Canada and the larger European countries (many of the smaller European countries effectively avoid the expense of implementing a PSI by following the lead of either one larger country or a basket of these). However, it is highly doubtful that any such differences can solely be attributed to the presence or absence of a PSI as the US differs from other rich country health systems on multiple dimensions of system structure and political economy (e.g., health funding models and governance structure).

### Summary of modelling approach

The approach we take in this paper is a simulation approach, in which we simulated a random collection of healthcare interventions. We then model two alternative scenarios in which decisions on funding these interventions are made – one scenario in which a PSI has been established and decides which interventions should be funded, and a counterfactual scenario in which there is no PSI and funding decisions are made on some other basis. For consistency, we will refer to these two funding scenarios as “PSI-active” and “PSI-absent”, respectively. The performance of each funding scenario is then compared according to financial and health-based metrics.

To represent the PSI-active scenario, we assume that the core PSI feature influencing funding decisions is the ability to conduct cost-effectiveness studies for a given intervention. Furthermore, we assume that only a PSI can provide such information in order to make decisions on this basis. We therefore model funding decisions in the PSI-active scenario as being based on a funding rule centred on a minimum threshold for the cost-effectiveness ratio (CER). To represent the counterfactual PSI-absent scenario, we assume that there is no access to cost-effectiveness information, and that funding decisions are reached independently of this information. We model the CER-independence of the counterfactual decisions by funding interventions on a first-come-first-served (FCFS) basis (that is, according to the random order in which interventions are sampled).

We recognise that, in practice, a PSI-absent scenario could avoid the effort and expense of explicitly setting priorities, yet still perform substantially better than funding interventions with a random collection of cost-effectiveness. We anticipate, however, that many counter-examples will in fact feature some degree of random selection, CER information (at some level), or a combination of these. For example, funding decisions on the basis of burden of illness would prioritise interventions which target the most substantial health gains (per individual or across a population). In the absence of information on the health gains that are actually delivered, however, this strategy can be assumed to fund interventions which achieve a random collection of CERs. Another counter-example is to fund interventions based on common-sense or expert knowledge, however, it could be expected that either of these would in fact draw upon some level of cost-effectiveness information – potentially from a PSI in another country. Indeed smaller PSIs may operate effectively by following CER advice from an external PSI; however this does not enable local characteristics to be incorporated into the decision-making process. Therefore, while we recognise that our modelling approach is obviously idealised, we believe that using a counterfactual setting which represents the true absence of information on CERs gives a new perspective on the question of the value of a PSI.

Furthermore, we recognise that the CER-threshold funding rule is an idealised implementation of the funding decisions that are made by PSIs in practice. We therefore consider various extensions to the CER-threshold funding rule, that are intended to more closely model aspects of how a PSI would operate in reality. These extensions broadly relate to budgetary considerations, and include considering: the total budget available for funding, focusing the funding on those interventions which will have the largest budgetary impact, and growing the available budget through time as the PSI becomes more established.

Specifically, we conduct the comparison of the PSI-active scenario against the PSI-absent scenario through four investigations – each with a different decision rule under which a PSI might operate, and an appropriate counterfactual decision rule (see Table 1). Case (i) reflects a PSI which operates using a cost-effectiveness threshold as its decision rule (somewhat similar to NICE in England). Case (ii) models a PSI which uses both cost-effectiveness and budget impact in its decision making (similar to HITAP in Thailand). Case (iii) adds some practicality to the previous case, since typically not all new technologies are subject to formal analysis: only those with the largest health or financial footprint are. For this Case, the number of technologies subject to formal analysis is assumed to be static, unchanging from year to year. In contrast, Case (iv) models the situation where a new PSI has been implemented, analysing an increasing number of technologies each year. The final column of Table 1 cross-refers to the mathematical expression of the decision rules deployed under the PSI-active and PSI-absent scenarios, as defined in Section 3.

**Table 1:** Summary of the comparison investigations analysed in Section 4

Name of case	Description of PSI rule	Description of counterfactual rule	Corresponding equations (refer to Section 3)
(i) Threshold rule	Fund interventions with cost-effectiveness ratio below the threshold	Fund random selection of interventions	(5) and (3)
(ii) Threshold rule with budget constraint	Fund interventions with cost-effectiveness ratio below the threshold and within the limited budget	Fund random selection of interventions within limited budget	(6) and (4)
(iii) Threshold rule with limited analysis capacity	Fund interventions with cost-effectiveness ratio below the threshold within the limited budget and with static limit on application of threshold rule	Fund random selection of interventions within limited budget	(7) and (4)
(iv) Threshold rule with phased run-in	Fund interventions with cost-effectiveness ratio below the threshold within the limited budget and with limit on application of threshold rule relaxing over time	Fund random selection of interventions within limited budget	(8) and (4)

We structure our findings by presenting analysis first for two base case data-sets, based on available data for the UK and Malawi. As our simulation is a stochastic simulation, analysis of these base cases allows us to explore the distribution of outcomes for fixed parameters. We follow this up by presenting sensitivity analyses (Appendix 4) which allow us to show how expected increases in benefit or cost savings vary depending on the cost-effectiveness threshold or the budget constraint used in the decision rules. An important driver of the results is the level of correlation between the costs and the benefits at the level of the interventions and we use the sensitivity analysis to explore that as well.

### Review of relevant literature for the modelling approach

To the best of our knowledge, there have not previously been any published works utilising economic modelling to quantify the contribution of a PSI. The simulation model presented here implements a portfolio decision analysis (PDA) framing to the PSI decision problem. PDA is a collection of methods, where the focus is on constructing a portfolio of “projects” from a larger pool, and the chosen portfolio is optimal with respect to one or more criteria. Typically a number of constraints restrict the projects which can be included in the portfolio, such as limited resources. Salo et al. [25] present a useful overview of PDA, with discussion of the underpinning theory and of a diverse range of applications. With two criteria – such as cost and benefit – the PDA problem equates to selecting those projects which present the best value for money. Phillips and e Costa [22] also provide a useful introduction to PDA and value-for-money project selection for two criteria, with discussion of the distinction between resource allocation and resource prioritisation. Morton et al. [21] present a PDA model for portfolio selection against multiple criteria, and also provide a survey of applications of PDA. Recent examples applying PDA in a variety of settings include prioritising strategic ecological interventions to monitor or manage different species or habitats [3], prioritising safety measures to avoid system failures [16, 17], prioritising the portfolio of monitoring systems to improve the reliability of power transmission networks [4], prioritising siting locations for offshore wind farms [5], prioritising maintenance programmes for bridges [18], and prioritising research and development funding for national energy programmes [15].

The approach taken here is inspired by the approach introduced by Keisler [13], who applied simulation in a PDA setting to explore the impact that additional information on a project’s uncertain value has on the value of the selected portfolio. Several alternative selection criteria were considered, and these were compared with a random selection decision, akin to our counterfactual, PSI-absent, FCFS decision rule. In our setting, the candidate healthcare interventions represent the “projects” from which a portfolio must be selected, the funding budget (when incorporated within a decision rule) represents the available resource, the criteria by which interventions are measured are the implementation cost and the health benefit gain, and the decision to be optimised is the composition of the portfolio of funded interventions. Finally, we observe that in the commentary by Angelis et al. [2] on methods to integrate alternative measures of benefit provided by a healthcare intervention when assessing the value of these in a health technology assessment (HTA) context, there is recognition that PDA would be a useful tool to maximise the benefits gained from a selection of these. This paper presents a first attempt to explore this, with consideration of the practical issues which arise when formulating the PSI-active funding decision rules, based on cost-effectiveness.

## Methodology

The simulation model is intended to represent the decision-making process that a funding body, such as a national health department, would undertake in each funding cycle in order to identify which health interventions should be allocated funding. The individual interventions are selected from a collection of potential candidates, and are characterised by the size of the population they would impact, as well as the costs and expected health benefits per case. In each simulation a new collection of candidate interventions is randomly generated. For each funding scenario – PSI-active and PSI-absent – the relevant funding decision rule (as defined in Table 1) is then applied to this collection, and the portfolio of interventions selected for funding under each scenario can be compared. This is repeated over many simulations, generating many random collections of candidate interventions, and producing a distribution of outcomes under each funding scenario.

Mathematically speaking, we model the set of *N* candidate interventions in a given simulation of a funding cycle (of fixed length) as ${I}_{A}=\{{I}_{1},{I}_{2},\ldots ,{I}_{N}\}$; the $i$ th intervention is defined as the tuple of random variables ${I}_{i}=({P}_{i},{C}_{i},{Q}_{i})$, where ${P}_{i}$ represents the size of the population impacted by the intervention, and the costs and expected health benefits per case that is allocated this intervention are represented by ${C}_{i}$ and ${Q}_{i}$, respectively.

To explore the value which could potentially be returned from a PSI, we consider the cases of two countries at different stages of establishing universal healthcare (UHC) systems: one country with well-established UHC (the UK) and one country with more fledgling UHC (Malawi). We use published data on healthcare interventions in the UK [10] and Malawi [23], respectively, to represent these two countries. Note that we only use these data-sets to provide indicative descriptions of the type of interventions which would be considered for funding in established UHC and fledgling UHC countries. We do not assume that the interventions described in these data-sets are fully representative of the specific funding decisions made in either the UK or Malawi. Furthermore, we do not assume that the results presented in the following section specifically represent the outcomes which could be expected from a PSI in either the UK or Malawi. The data-sets used are presented in Appendix 1 for clarity.

From the outset, the intention has been to provide an online release of the simulation model to facilitate dissemination to end-users, and that this should be technically accessible for non-quantitative users. Intended end-users include health policy makers and funders considering establishing PSIs, who would benefit from gaining an understanding of the potential value a PSI could deliver in comparison to existing approaches to fund health technology interventions, as well as the potential impact of different approaches by which a PSI makes those decisions. Additionally, end-users could include directors of established PSIs, who would benefit from being able to estimate the value that the institution delivers to a national health programme. For example, Kingkaew et al. [14] present an application of the simulation model to the case of Thailand, to demonstrate the positive impact that HTA in Thailand has had on national health expenditure. This end-user consideration has driven various modelling choices, in particular the distributional form of the input data, and the dependency modelling of the output data.

Following a preliminary analysis of the indicative data-sets (see Appendix 3 for details), the number of cases treated by each intervention, the incremental costs of administering each intervention per treated case, and the incremental health benefits returned per case treated with the intervention, are each assumed to be log-normally distributed, and are specified respectively as $P\sim LogN({\mu }_{P},{{\sigma }_{P}}^{2})$, $C\sim LogN({\mu }_{C},{{\sigma }_{C}}^{2})$ and $Q\sim LogN({\mu }_{Q},{{\sigma }_{Q}}^{2})$, where $\mu$ and $\sigma$ represent the mean and standard deviation of each distribution, and the subscripts correspond to the respective random variables. These stochastic model parameters are summarised in Table 3, and the distribution parameters and other inputs are defined in Table 2.

**Table 2:** Input parameters used for the Section 4 investigations

Intervention measure	Country data-set	Relevant data for calculation and source	Symbol	Input parameter value
Intervention QALY increment	UK	${Q}_{i}$ per intervention, as shown in Table 9 and extracted from Guthrie et al. [10]	${\mu }_{Q}$ (Mean)	0.62
			${\sigma }_{Q}$ (Std. dev.)	1.18
	Malawi	${QT}_{i}/{P}_{i}$ per intervention, each as shown in Table 10 and extracted from Ochalek et al. [23]	${\mu }_{Q}$ (Mean)	7.05
			${\sigma }_{Q}$ (Std. dev.)	16.93
Intervention cost increment	UK	${C}_{i}$ per intervention, as shown in Table 9 and extracted from Guthrie et al. [10]	${\mu }_{C}$ (Mean)	£9.14k
			${\sigma }_{C}$ (Std. dev.)	£19.05k
	Malawi	${CT}_{i}/{P}_{i}$ per intervention, each as shown in Table 10 and extracted from Ochalek et al. [23]	${\mu }_{C}$ (Mean)	$\$20$
			${\sigma }_{C}$ (Std. dev.)	$\$30$
Number of cases per intervention (thousands)	UK	${P}_{i}$ per intervention, as shown in Table 9 and extracted from Guthrie et al. [10]	${\mu }_{P}$ (Mean)	540.22
			${\sigma }_{P}$ (Std. dev.)	595.41
	Malawi	${P}_{i}$ per intervention, as shown in Table 10 and extracted from Ochalek et al. [23]	${\mu }_{P}$ (Mean)	1464.74
			${\sigma }_{P}$ (Std. dev.)	2910.67
Number of interventions considered for funding	UK	Taken from Guthrie et al. [10], see Appendix 1	$N$	74
	Malawi	See Appendix 1		40
CER threshold required for funding	UK	Taken from Guthrie et al. [10], see Appendix 1	$t$	£20k
	Malawi	See Appendix 1		$\$2$
Annual budget limit to fund interventions	UK	See Appendix 1	$l$	£75,000M
	Malawi			$\$120\mathrm{M}$
Number of interventions funded annually	UK		$n$	48
	Malawi			14
Annual percentage of high-value interventions assessed using the threshold	Both		${p}_{a}$	$20\%$
Total number of years of phased increases to the PSI capability			$Y$	$5\ \text{Years}$
Target percentage of interventions assessed by the PSI in the final year of phased increases			${p}_{Y}$	$100\%$

**Table 3:** Stochastic model parameters (describing each intervention within each simulation) used for the Section 4 and Appendix 4 investigations

Sampled components of $i$ th intervention (*I*_*i*_)	Symbol	Distribution	Parameterisation
QALY increment of $i$ th intervention	${Q}_{i}$	Log-normal	${Q}_{i}\sim LogN({\mu }_{Q},{{\sigma }_{Q}}^{2})$
Cost increment of $i$ th intervention	${C}_{i}$	Log-normal	${C}_{i}\sim LogN({\mu }_{C},{{\sigma }_{C}}^{2})$
Number of cases impacted by $i$ th intervention	${P}_{i}$	Log-normal	${P}_{i}\sim LogN({\mu }_{P},{{\sigma }_{P}}^{2})$

The simulation model incorporates statistical dependence between costs and benefits by generating samples which have a specified level of linear correlation, which we define as ${\rho }_{CQ}$. The details of the approach to achieve correlated samples is outlined in Appendix 2. For a correlation close to one, the costs and benefits of an intervention will have a strong linear dependence, and the most expensive interventions are more likely to deliver the largest health benefit gains. In contrast, for a correlation close to zero, the costs and benefits will be largely independent and the most expensive interventions will be as likely to deliver large or small health benefit gains. For reference, the costs and benefits for the indicative data-sets (see Appendix 1) have a correlation of 0.164 for Malawi and 0.998 for the UK. Statistical dependence is a somewhat abstract concept, and would be challenging for many users to quantify or accurately measure without a significant amount of data. To mitigate this, the simulation model therefore automatically runs under three different levels of correlation: a low correlation ${(\rho }_{CQ}=0.2)$ where the costs and benefits are largely independent, a medium correlation ${(\rho }_{CQ}=0.5)$ where there is some dependence between costs and benefits, and a high correlation $(\rho_{CQ}=0.8)$ where the costs and benefits have a strong dependence. Note that the correlation values ${\rho }_{CQ}=\{{0.2,0.5,0.8}\}$ specify the correlation between the untransformed costs and benefits. This provides a simple mechanism to demonstrate to users the potential impact of dependency between costs and benefits in terms of the model outputs.

Application of each funding decision rule *D *yields a subset of ${I}_{A}$, comprising those interventions which satisfy the particular funding requirements of the decision rule. Formally these are written as

1 $${I}_{CF}=\{I\in {I}_{A} | {D}^{C}\left(I\right)=1\} ,$$

for the portfolio of interventions funded through counterfactual PSI-absent decision rule *D*^*C*^, and

2 $${I}_{TH}=\{I\in {I}_{A} | {D}^{T}\left(I\right)=1\},$$

for the portfolio of interventions funded through the CER-threshold-based PSI-active decision rule ${D}^{T}$. Below, we investigate and compare several variants of the PSI-absent and PSI-active decision rules, in order to explore the performance of the PSI-active funding scenario under various alternative approaches to implement a PSI in practice.

Two counterfactual PSI-absent decision rules are considered. Firstly, a count-based FCFS rule, defined for the $i$ th intervention as

3 $$D_C^C\left(I_i,\;n\right)=\left\{\begin{array}{c}1\;\text{if}\;i\leq n,\;\text{for}\;n\in N\vert n\leq N,\\0\;\text{otherwise},\end{array}\right.$$

where *n* represents the number of interventions funded. Secondly, a budget-based FCFS rule, defined for the $i$ th intervention as

4 $$D_B^C\left(I_i,\;l\right)=\left\{\begin{array}{c}1\ if \overset{i}{\underset{j=1}{\ \sum\;}}C_j\leq l,{\text {for}}\ l\in R,\\0\ {\text {otherwise}},\end{array}\right.$$

where $l$ represents a budget limit.

The simplest PSI-active decision rule consists of a requirement to meet a CER threshold, defined for the $i$ th intervention as

5 $$D_R^T\left(I_i\mathit,\mathit\;t\right)=\left\{\begin{array}{c}1\;\text{if}\frac{C_i}{Q_i}\mathit\leq\;t\mathit,\;\text{for}\;t\in\mathrm R,\\0\;\text{otherwise},\end{array}\right.$$

where $t$ represents the threshold. This rule is then iteratively increased in complexity. Firstly, incorporating a budget limit $l$ gives for the $i$ th intervention

6 $$D_B^T\left(I_i,t,l\right)=\left\{\begin{array}{c}1\;\text{if}\frac{C_i}{Q_i}\leq t,\sum_{j=1}^iC_j\leq l,\text{for}\;t,l\in R,\\0\;\text{otherwise}.\end{array}\right.$$

An alternative PSI-active rule is that the cost-effectiveness analysis would only be applied to a limited number of interventions – specifically those interventions which would be the most expensive to implement. The motivation for this rule is that the PSI has limited resources, and therefore has to be selective in which interventions require review. A natural approach to this scenario would therefore be to focus on those interventions which will have the largest impact on the available funding and apply the CER threshold decision rule to these interventions. All other interventions are simply funded according to the counterfactual rule on a FCFS basis. For the *i*^th^ intervention this rule is defined as

7 $$D_P^T\left(I_i,t,l,p_a\right)=\left\{\begin{array}{c}1\;\text{if}\;P_iC_i\geq{P_p}_a{C_p}_a,\sum_{j=1}^iC_j\leq l,\frac{C_i}{Q_i}\leq t,\;\text{for}\;t,l,p_a\in R,0\leq p_a\leq100,\\1\;\text{if}\;P_iC_i<{P_p}_a{C_p}_a,\sum_{j=1}^iC_j\leq l,\;\text{for}\;l,p_a\in R,0\leq p_a\leq100,\\0\;\text{otherwise},\end{array}\right.$$

where ${{P}_{p}}_{a}{{C}_{p}}_{a}$ is the $(100-{p}_{a})$ th percentile of the set ${\{P}_{1}{C}_{1},{P}_{2}{C}_{2},\ldots ,{P}_{N}{C}_{N}\}$. The decision parameter ${p}_{a}$ therefore sets the annual proportion of highest budget-impacting interventions that a CER assessment is applied to. A final alternative PSI-active rule is to implement a phased increase of the PSI capability, such that over a number of years the PSI will gradually increase the proportion of interventions which are reviewed, until a mature state of operation is achieved. The motivation for this addition is that the expertise, resourcing and funding for a PSI may be increased over time as the institute establishes itself. As such, it is only possible to review a portion of all interventions which seek funding, and in a similar approach to the previous rule, the focus is placed on those interventions which will have the largest impact on the available funding budget. For lower-value interventions, the funding decision is again determined by a budget-based FCFS rule. For the *i*^th^ intervention this rule is defined as

8 $${D}_Y^T\left({I}_i,t,l,{p}_Y,y,Y\right)=\left\{\begin{array}{c}1\ \text{if}\ {P}_i{C}_i\geq {P}_r{C}_r,\sum_{j=1}^i{C}_j\leq l,\frac{C_i}{Q_i}\leq t,\kern0.5em \text{where}\kern0.5em r=\frac{p_Yy}{Y},\text{for}\ t,l,{p}_Y\in R,y,Y\in N,0\leq {p}_Y\leq 100,1\leq y\leq Y,\\ {}1\ \text{if}\ {P}_i{C}_i<{P}_r{C}_r,\sum_{j=1}^i{C}_j\leq l,\text{where}\ r=\frac{p_Yy}{Y},\text{for}\ l,{p}_Y\in R,y,Y\in N,0\leq {p}_Y\leq 100,1\leq y\leq Y,\\ {}0\ \text{otherwise},\end{array}\right.$$

where ${P}_{r}{C}_{r}$ is the $(100-r)$ th percentile of the set ${\{P}_{1}{C}_{1},{P}_{2}{C}_{2},\ldots ,{P}_{N}{C}_{N}\}$, $Y$ represents the total number of years of phasing, and $y$ represents the current year within this phased-approach. Defining *r *in this way controls the percentage of interventions each year which are funded based on a CER threshold decision. In the first year the ${p}_{Y}/Y \%$ of interventions with largest $P\times C$ are funded based on a CER threshold decision, and in year $Y$ this has increased to the largest ${p}_{Y}$% of interventions.

Intervention portfolio ${I}_{CF}$ is determined through Eq. (1), with the counterfactual PSI-absent decision rule ${D}^{C}={D}_{C}^{C}$ or ${D}^{C}={D}_{B}^{C}$, as given by Eqs. (3)-(4). Similarly, portfolio ${I}_{TH}$ is determined through Eq. (2), with the PSI-active decision rule either ${D}^{T}={D}_{R}^{T}$, ${D}^{T}={D}_{B}^{T}$, ${D}^{T}={D}_{P}^{T}$, or ${D}^{T}={D}_{Y}^{T}$, as given by Eqs. (5)-(8). Each simulation generates a new set of candidate interventions ${I}_{A}$, and thus new portfolios ${I}_{CF}$ and ${I}_{TH}$. The decision rules Eq. (4) - Eq. (8) correspond respectively to the Case (i) - Case (iv) investigations, as summarised in Table 1.

In a given simulation, various metrics can be utilised to compare portfolios ${I}_{CF}$ and ${I}_{TH}$, in order to communicate the differences between implementing the PSI-absent and PSI-active decision rules across the simulations. The metrics used here are largely built upon two key measures: the difference in total costs between all interventions in each portfolio, and the difference in total benefits between all interventions in each portfolio. These differences are formally defined as

9 $${\Delta }_{C}\left( {I}_{TH},{I}_{CF}\right)= \sum_{{I}_{i}\in {I}_{TH}}{C}_{i}- \sum_{{I}_{i}\in {I}_{CF}}{C}_{i},$$

and

10 $${\Delta }_{Q}\left( {I}_{TH},{I}_{CF}\right)= \sum_{{I}_{i}\in {I}_{TH}}{Q}_{i}- \sum_{{I}_{i}\in {I}_{CF}}{Q}_{i},$$

respectively. The additional metrics used in Section 4 which follow from these, are the net health benefit (NHB), defined for CER threshold $t$ as

11 $${NHB({I}_{TH},I}_{CF},t)={\Delta }_{Q}\left( {I}_{TH},{I}_{CF}\right)- \frac{1}{t}{\Delta }_{C}\left( {I}_{TH},{I}_{CF}\right),$$

and the incremental cost-effectiveness ratio (ICER), defined as

12 $${ICER({I}_{TH}, I}_{CF})=\frac{{\Delta }_{C}\left( {I}_{TH},{I}_{CF}\right)}{{\Delta }_{Q}\left( {I}_{TH},{I}_{CF}\right)}$$

The model outputs communicate the differences between implementing the PSI-absent and PSI-active decision rules across the simulations. Costs and benefits are presented at a population level per funding cycle, and the impact on these is explored separately and also in combination.

The input parameters used for the investigations on the indicative UK and Malawi data-sets are presented in Table 2, along with details of sources and calculations as relevant. Table 3 completes the parameterisation of the decision rules (3)-(8), with details of the stochastic model parameters and how these relate to the inputs.

To summarise the modelling process, first the comparison investigation is selected (Case (i) – (iv) as detailed in Table 1). This determines which of the funding decision rules (3)-(8) are applied for the PSI-active and PSI-absent funding scenarios. The parameters for the relevant decision rules are fixed according to Table 2. For each comparison investigation, 1000 simulation runs are completed. In each simulation the set of candidate interventions ${(I}_{A})$ are sampled, according to the sampling parameters defined in Table 3 and relevant distribution input parameters from Table 2. The relevant decision rules are applied to the set of interventions ${I}_{A}$, and various output performance measures (including Eqs. (9)-(12)) are calculated. At the next simulation, a new set of interventions is sampled and the process repeats. The intervention components $Q$, $C$ and $P$ (see Table 3) are the only stochastic parameters, varying between simulations.

## Results for the base case

The four investigations outlined in Table 1 are presented in Sections 4.1–4.4 respectively. Appendix 4 presents additional analysis on these investigations, as the parameters of the PSI-active decision rules are varied.

### Case (i): Performance of threshold rule

The simulation model was run on each of the indicative data-sets with 1000 simulation runs. Table 4 shows a summary comparison of the outputs generated for the Case (i) investigation under the PSI-absent and PSI-active funding scenarios (see Table 1 for definitions and Table 2 for parameterisations of the decision rules). Comparing both scenarios at a particular level of correlation for either data-set reveals that the PSI-active scenario will spend less funding, will deliver higher health benefits, will achieve a lower ICER (– comparing to a situation before the funding is spent), and will fund more interventions.

**Table 4:** Summary results from the Case (i) simulations comparing the PSI-absent (Absent) and PSI-active (Active) funding scenarios. Input data is parameterised from the indicative UK and Malawi data-sets as presented in Appendix 1. For each performance measure and each correlation level, the best performing scenario is marked in bold

Indicative data-set	Output measure	Correlation	Funding approach	
			Absent	Active
UK	Average total cost (£M)	0.2	237,772	**115,182**
		0.5	229,362	**151,748**
		0.8	235,271	**215,584**
	Average total QALY (thousands)	0.2	16,444	**20,759**
		0.5	15,713	**19,475**
		0.8	16,034	**19,571**
	Average number of interventions funded	0.2	**48**	44.58
		0.5	**48**	47.6
		0.8	48	**54.27**
	Expected ICER per funding cycle	0.2	15.75	**5.78**
		0.5	15.24	**7.93**
		0.8	14.88	**11.05**
	Expected NHB of PSI-active scenario compared to PSI-absent scenario (thousand QALYs per funding cycle)	0.2	**10,445**	
		0.5	**7,643**	
		0.8	**4,522**	
Malawi	Average total cost ($M)	0.2	333	**192**
		0.5	333	**241**
		0.8	339	**289**
	Average total DALY (thousands)	0.2	146,278	**299,245**
		0.5	144,431	**265,199**
		0.8	151,685	**223,009**
	Average number of interventions funded	0.2	14	**15.51**
		0.5	**14**	13.64
		0.8	**14**	10.03
	Expected ICER per funding cycle	0.2	0.0032	**0.0007**
		0.5	0.0029	**0.001**
		0.8	0.0026	**0.0014**
	Expected NHB of PSI-active scenario compared to PSI-absent scenario (thousand DALYs per funding cycle)	0.2	**223,483**	
		0.5	**166,718**	
		0.8	**96,028**	

Comparing results across different levels of correlation, it is clear that with higher correlation the performance of the PSI-active scenario reduces. This reduction is due to the fact that with higher correlation levels the health benefits are more proportional to the costs. While this largely mitigates the risk of funding an expensive intervention which will deliver little health benefit, it also diminishes the opportunities for funding low cost interventions which deliver substantial benefits. Even with higher correlation, however, it is clear that the PSI-active funding scenario is still superior to the PSI-absent scenario. For brevity, only the mid-level case with correlation equal to 0.5 will be analysed in the remainder of this section. This provides a comparison between the PSI-active and PSI-absent funding scenarios when there is some dependence between the intervention costs and benefits – if that dependence reduces then the performance of the PSI-active scenario improves, and if that dependence increases then the performance of the PSI-active scenario degrades. Note that the Appendix 4 sensitivity analysis provides further comparison between the correlation levels as the decision rules are varied.

Comparing between data-sets, the indicative Malawi data shows larger benefits to be gained under the PSI-active scenario. The NHB represents a larger percentage of the average total health benefit returned under each level of correlation, and even with high correlation between costs and benefits the expected ICER for the PSI-active scenario is only 54% of that for the PSI-absent scenario.

**Fig. 1:**
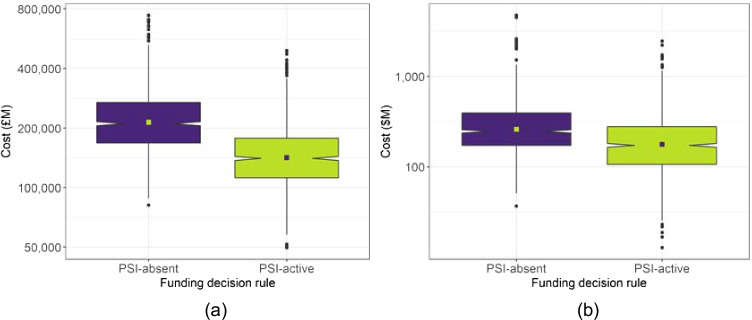
Distribution of total incremental expenditure per funding cycle for each decision rule. (**a**) Indicative UK data, (**b**) Indicative Malawi data

**Fig. 2:**
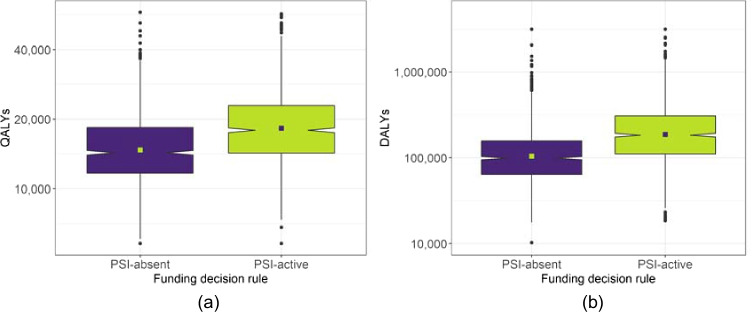
Distribution of total incremental QALY gain per funding cycle for each decision rule. (**a**) Indicative UK data, (**b**) Indicative Malawi data

The total expenditure per funding cycle is displayed in Fig. 1 for each funding scenario on each indicative data-set. The distribution of results across all simulations are summarised as a box-plot, with the central notches on each box-plot representing a confidence interval around the median. The contrasting square on each box-plot represents the mean. Similarly, Fig. 2 shows the distribution across simulations of the total health benefit per funding cycle for each funding scenario on each indicative data-set.

The findings discussed above for Table 4 are portrayed in more detail in Figs. 1 and 2 for the total costs and benefits, respectively. In each case, and across both data-sets, the data are shown to have a relatively focused distribution in terms of the overall range of each variable. The median total cost is slightly higher for the PSI-absent scenario with both data-sets, and the median total QALY gain is higher for the PSI-active scenario with both data-sets.

**Fig. 3:**
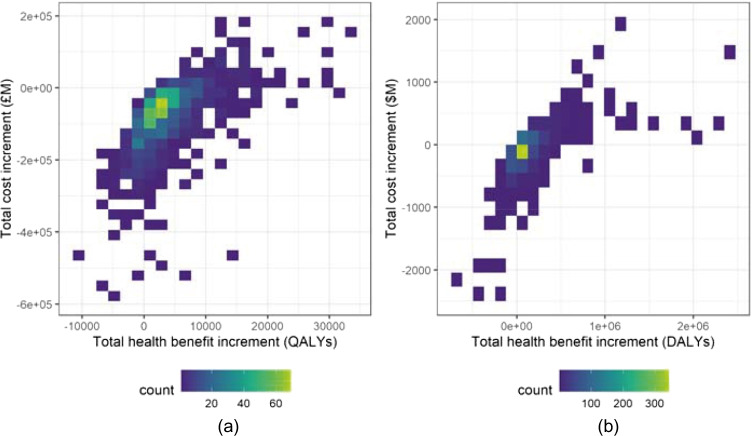
Distribution of the difference in the total cost of funded interventions (Eq. (9)) against the difference in the total health benefit gain of funded interventions (Eq. (10)), per funding cycle for each decision rule. (**a**) Indicative UK data, (**b**) Indicative Malawi data

Figure 3 plots the differences ${\Delta }_{Q}\left( {I}_{TH},{I}_{CF}\right)$ in each simulation against the corresponding differences ${\Delta }_{C}\left( {I}_{TH},{I}_{CF}\right)$ across all simulations. It is clear that the majority of recorded differences for each data-set demonstrate an increase in health benefits under the PSI-active scenario, in addition to a decrease in implementation costs. Figure 3 shows that the largest mass for each bi-variate distribution is focused on slightly reduced costs and slightly improved health benefits, although the distribution for each data-set is reasonably spread. An important observation is that, although some simulations demonstrate that under the PSI-active scenario the benefits increased but required more funding, and in a few cases the benefits reduced but required less funding, there are no cases where costs increase and benefits decrease when comparing the PSI-active scenario to the PSI-absent scenario. This is demonstrated further in Table 5, which classifies simulations according to whether the cost- and health benefit increments are positive or negative.

**Table 5:** Classification of the total cost increment (Eq. (9)) and the total health benefit increment (Eq. (10)) per simulation for the Case (i) investigation, for the indicative UK and Malawi data-sets. For each data-set, the percentage of simulations with a positive cost increment (that is, the funded interventions are more expensive in the PSI-active scenario) and also a negative health benefit increment (that is, the funded interventions produce fewer health benefits in the PSI-active scenario) is marked in bold

	Classification	${\Delta }_{Q}\left( {I}_{TH},{I}_{CF}\right)<0$(%)	${\Delta }_{Q}\left( {I}_{TH},{I}_{CF}\right)\geq 0$(%)	Totals (%)
UK	${\Delta }_{C}\left( {I}_{TH},{I}_{CF}\right)\geq 0$(%)	**0**	11.2	11.2
	${\Delta }_{C}\left( {I}_{TH},{I}_{CF}\right)<0$(%)	16	72.8	88.8
	Totals (%)	16	84	100
Malawi	${\Delta }_{C}\left( {I}_{TH},{I}_{CF}\right)\geq 0$(%)	**0**	31.7	31.7
	${\Delta }_{C}\left( {I}_{TH},{I}_{CF}\right)<0$(%)	18.3	50	68.3
	Totals (%)	18.3	81.7	100

### Case (ii): Performance of threshold rule with budget constraint

**Fig. 4:**
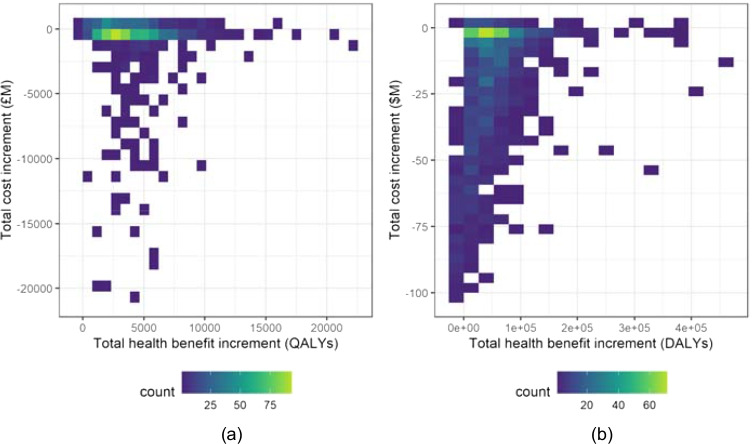
Distribution of the difference in the total cost of funded interventions (Eq. (9)) against the difference in the total health benefit gain of funded interventions (Eq. (10)), per funding cycle for each decision rule, with budget limits. (**a**) Indicative UK data, (**b**) Indicative Malawi data

The impact of the budget restricted decision rules (4) and (6) (for the PSI-active and PSI-absent scenarios, respectively) is displayed in Fig. 4. There is a much greater performance difference between the two data-sets in this case. For the indicative Malawi data, the largest concentration of the data is once again at slight reductions in costs with slight increases in benefits, however, the differences within each simulation have a more disperse distribution than for the Case (i) decision rules. For the indicative UK data, the largest concentration of the data is also at slightly increased benefits, with the cost differences spread between slight increases and slight reductions. There are, however, a small number of simulations which are shown to result in increased costs and reduced benefits. Further exploration of these cases reveals that these outcomes are caused by lost funding opportunities, as a result of applying the funding decision rules on a case-by-case basis with random ordering of interventions. Consider for example each funding approach being applied to a particular list of interventions, and the PSI-active scenario has insufficient budget remaining to fund a specific (very low CER) intervention; in contrast, under the PSI-absent scenario, previous funding decisions have resulted in a larger budget remaining. The low CER intervention is therefore funded, and as a result the PSI-absent scenario achieves lower overall costs and higher health benefit. Table 6 highlights that this only applies to a minimal number of simulations for either data-set, and that the vast majority of simulations result in improved health benefits under the PSI-active scenario, and with also reduced costs in the majority of simulations.

**Table 6:** Classification of the total cost increment (Eq. (9)) and the total health benefit increment (Eq. (10)) per simulation for the Case (ii) investigation, for the indicative UK and Malawi data-sets. For each data-set, the percentage of simulations with a positive cost increment (that is, the funded interventions are more expensive in the PSI-active scenario) and also a negative health benefit increment (that is, the funded interventions produce fewer health benefits in the PSI-active scenario) is marked in bold

	Classification	${\Delta }_{Q}\left( {I}_{TH},{I}_{CF}\right)<0$(%)	${\Delta }_{Q}\left( {I}_{TH},{I}_{CF}\right)\geq 0$(%)	Totals (%)
UK	${\Delta }_{C}\left( {I}_{TH},{I}_{CF}\right)\geq 0$(%)	**0.2**	25.5	25.7
	${\Delta }_{C}\left( {I}_{TH},{I}_{CF}\right)<0$(%)	0.3	74	74.3
	Totals (%)	0.5	99.5	100
Malawi	${\Delta }_{C}\left( {I}_{TH},{I}_{CF}\right)\geq 0$(%)	**0.1**	4.7	4.8
	${\Delta }_{C}\left( {I}_{TH},{I}_{CF}\right)<0$(%)	7.3	87.9	95.2
	Totals (%)	7.4	92.6	100

### Case (iii): Performance of threshold rule with limited analysis capacity

**Fig. 5:**
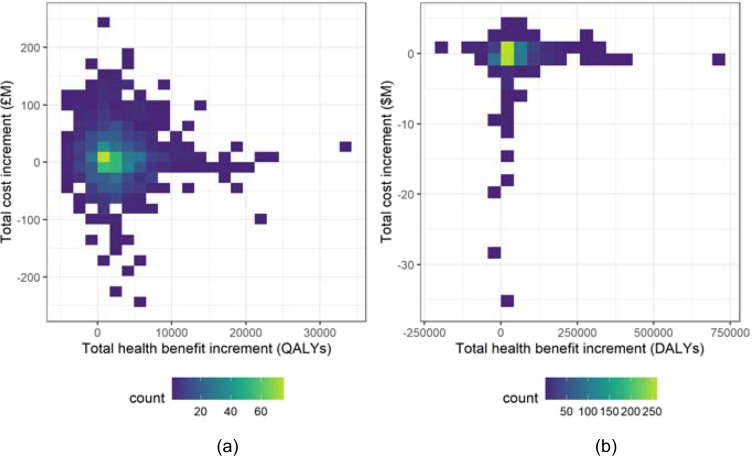
Distribution of the difference in the total cost of funded interventions (Eq. (9)) against the difference in the total health benefit gain of funded interventions (Eq. (10)), per funding cycle for each decision rule, with limited application of threshold rule. (**a**) Indicative UK data, (**b**) Indicative Malawi data

Figure 5 explores the performance of the PSI-active funding rule (7) against the PSI-absent rule (4). Again, there are substantial differences in performance between both data-sets. For the indicative UK data, the differences between the funding scenarios is much more widely distributed. There are a substantial number of cases where costs increase and benefits decrease under the PSI-active scenario; however, the most frequently observed difference is shown to be slightly improved health benefits with larger increases to the implementation costs. For the indicative Malawi data, there is a reasonably disperse distribution of the observed differences between the two funding scenarios, however, the most frequently observed differences show slightly reduced costs for a range of health benefit improvements (Table 7).

**Table 7:** Classification of the total cost increment (Eq. (9)) and the total health benefit increment (Eq. (10)) per simulation for the Case (iii) investigation, for the indicative UK and Malawi data-sets. For each data-set, the percentage of simulations with a positive cost increment (that is, the funded interventions are more expensive in the PSI-active scenario) and also a negative health benefit increment (that is, the funded interventions produce fewer health benefits in the PSI-active scenario) is marked in bold

	Classification	${\Delta }_{Q}\left( {I}_{TH},{I}_{CF}\right)<0$(%)	${\Delta }_{Q}\left( {I}_{TH},{I}_{CF}\right)\geq 0$(%)	Totals (%)
UK	${\Delta }_{C}\left( {I}_{TH},{I}_{CF}\right)\geq 0$(%)	**10.6**	47.1	57.7
	${\Delta }_{C}\left( {I}_{TH},{I}_{CF}\right)<0$(%)	7	35.3	42.3
	Totals (%)	17.6	82.4	100
Malawi	${\Delta }_{C}\left( {I}_{TH},{I}_{CF}\right)\geq 0$(%)	**4**	42.4	46.4
	${\Delta }_{C}\left( {I}_{TH},{I}_{CF}\right)<0$(%)	9.6	44	53.6
	Totals (%)	13.6	86.4	100

### Case (iv): Performance of threshold rule with phased run-in

**Fig. 6:**
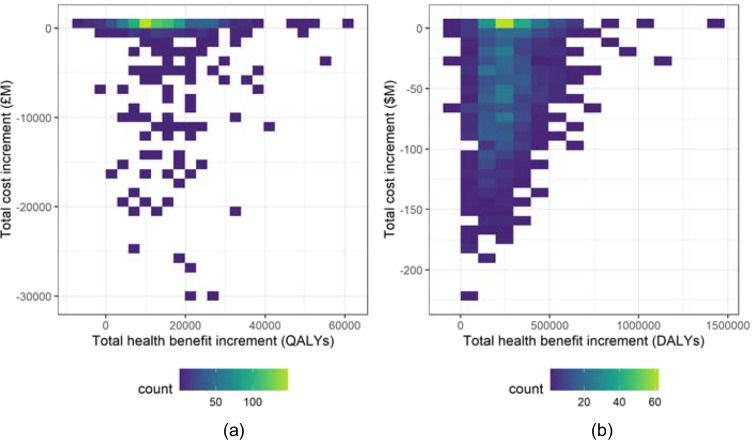
Distribution of the difference in the total cost of funded interventions (Eq. (9)) against the difference in the total health benefit gain of funded interventions (Eq. (10)), per funding cycle for each decision rule, with phased increase of the application of the threshold rule. (**a**) Indicative UK data, (**b**) Indicative Malawi data

Finally, the phased increase scenario (given by the PSI-active funding rule (8)) is considered, with $Y=5$ funding cycles and $p=100\%$ of interventions funded based on the CER threshold at the end of this period. The percentage of interventions reviewed by the PSI will therefore increase by 20% in each cycle over this period. The results from this comparison are shown in Fig. 6. For the indicative UK data, overall costs over this period are shown to typically increase, however, the health benefits which would be returned are also shown to increase substantially. For the indicative Malawi data, the most frequently observed result is that the overall costs reduce and overall benefits increase, and this difference is observed in the vast majority of cases (Table 8).

**Table 8:** Classification of the total cost increment (Eq. (9)) and the total health benefit increment (Eq. (10)) per simulation for the Case (iv) investigation, for the indicative UK and Malawi data-sets. For each data-set, the percentage of simulations with a positive cost increment (that is, the funded interventions are more expensive) and also a negative health benefit increment (that is, the funded interventions produce fewer health benefits) is marked in bold

	Classification	${\Delta }_{Q}\left( {I}_{TH},{I}_{CF}\right)<0$(%)	${\Delta }_{Q}\left( {I}_{TH},{I}_{CF}\right)\geq 0$(%)	Totals (%)
UK	${\Delta }_{C}\left( {I}_{TH},{I}_{CF}\right)\geq 0$(%)	**2.1**	80.3	82.4
	${\Delta }_{C}\left( {I}_{TH},{I}_{CF}\right)<0$(%)	0.3	17.3	17.6
	Totals (%)	2.4	97.6	100
Malawi	${\Delta }_{C}\left( {I}_{TH},{I}_{CF}\right)\geq 0$(%)	**0.1**	18.6	18.7
	${\Delta }_{C}\left( {I}_{TH},{I}_{CF}\right)<0$(%)	0.4	80.9	81.3
	Totals (%)	0.5	99.5	100

## Discussion

The investigations presented in Section 4 explore the potential impact of a PSI, when the mechanism by which the PSI reviews interventions is varied. An idealised scenario would be that any intervention which satisfies the cost-effectiveness ratio threshold would be funded (see Case (i) investigation). It is clear that under this approach substantial net savings would be realised, with a net surplus greater than the initial investment observed on average for both data-sets with a mid-level of correlation between the intervention costs and benefits.

In practice, however, it is likely that a funding budget will exist and that all interventions which satisfy the threshold rule will not necessarily be funded (see Case (ii) investigation). The implication of this is that inclusion of a budget limit implicitly introduces a FCFS nature to the threshold rule approach. As a consequence, there is potential that opportunity loss will result in a net loss when the threshold rule is compared with a budget-limited counterfactual rule. With both data-sets analysed in Section 4 these occurrences are found to be rare, and the expected scenario is that the threshold rule will still deliver substantial net savings. Restricting the PSI further such that the threshold rule is only applied to high-value interventions (see Case (iii) - Case (iv) investigations), the scale of each funding decision subjected to the threshold rule increases. The scale of net losses which can potentially occur through opportunity loss also therefore increases, and in Section 4 these losses are observed with a higher frequency for each data-set. Transitioning between these two funding approaches (from limited application of threshold rule to application of the threshold rule to all interventions), and the net surplus which would be returned by applying the threshold rule transitions accordingly.

The simple decision rules considered here may not accurately represent the full complexity of a PSI decision making process in practice. Indeed, for some PSIs decisions may not be driven by a threshold-based rule (see Millar et al. [19] for further discussion on this). The healthcare funding context modelled here is also a simplification. For example, in many LMICs, healthcare funding comes from national budgets as well as various other donor sources. Donors may target funding towards specific diseases and interventions according to their own agendas, rather than focusing specifically on the CER of interventions and making funding decisions on this basis. The modelling approach presented here could be employed separately by each funding source in order to prioritise its own intervention funding decisions – allowing for the potential that there is a prior down-selection of interventions according to external prioritisation considerations (see for example Glassman et al. [8] and Teerawattananon et al. [26] for practical examples of CER-driven decisions on this basis). At present, however, our approach would require development to support funding decisions across multiple funding sources.

The value of our modelling approach, however, is that estimates of the expected net benefits returned from a threshold rule can be quantified. This provides a clear normative benchmark for an idealised implementation of a PSI, and so the impact of any deviations from this implementation can also be quantified. Summarising the analysis of Section 4, either type of restriction to the application of the threshold rule is likely to result in a reduced net saving, and imposing any such restriction should be carefully assessed in terms of the justification for pursuing a less economically prudent approach. This quantified assessment therefore enables reasoned and evidence-based discussions on the mechanisms by which a PSI can implement intervention funding decisions.

The analysis that is set out in Section 4 and Appendix 4 is focused specifically on the portfolio of interventions (in terms of total costs and benefits) that would be funded under PSI-active and PSI-absent scenarios. That is, the focus is on the impact of the decision-making process in each scenario. A key consideration in order to fully address the title question of this paper (on the value of priority setting), is to account also for the cost of the decision-making process itself, under the PSI-active and PSI-absent scenarios. That is, to account for the additional cost in a PSI-active scenario of making each HTA. Glassman and Chalkidou [6] present an overview on the costs per HTA at a number of national PSIs. These range from $3,000 (as a lower limit in Uruguay) to $600,000 (as an upper limit in Germany). Using a current exchange rate, the upper limit for each UK HTA is approximately £300,000 ($400,000). Applying this cost to 74 HTAs per funding cycle for the UK data, this would comprise between 0.01% and 0.02% of the average total costs presented in Table 4. Using the Uruguay data as a proxy for the Malawi cost per HTA, then applying this cost to 40 HTAs per funding cycle would comprise between 0.04% and 0.06% of the average total costs presented in Table 4. While it is important to consider the additional HTA costs required to facilitate decision-making in a PSI-active scenario, this brief analysis therefore indicates that the potential for returned benefits would dwarf any decision-making costs. When applying our modelling approach to the case of Thailand, Kingkaew et al. [14] explicitly account for the additional cost for performing each HTA, and it is evident that these costs comprise less than 0.2% of the total cost estimates. An additional consideration is that the decision-making process in a PSI-absent scenario is not necessarily without cost itself, and the returned benefits estimated under the PSI-active scenario may therefore be conservative.

The threshold rule with limited analysis capacity (Case (iii) investigation, presented in Section 4.3) assumes that the available analysis would be deployed to evaluate those technologies which have the largest budgetary impact. In practice, however, the approach to deploying analysis may be more complex, and may include topic selection that identifies illness burdens to be prioritised for analysis. The pathway to scale-up the analytical capability then becomes more complex, and this is something we hope to investigate in future work. The final decision rule comparison presented in Section 4 (Case (iv) investigation, for a threshold rule with phased run-in) touches on the dynamic nature of the influence a PSI can make to intervention funding decisions. The full extent of this, however, is far more complex. Once an intervention is approved for funding, it will typically continue to be funded for many subsequent years, with any budget saving and health benefit gain realised year after year. The budget for intervention funding in a given year will therefore consist of funding for both newly approved and legacy interventions. Each year after the introduction of the PSI, the UHC system incrementally progresses towards a more sustainable and efficient state, in terms of the net savings accrued from cost reductions and monetised health benefit gains. The full influence of a PSI on annual intervention funding is therefore likely to be in a state of flux over a long period of time. This will only stabilise when the saturation of PSI-approved interventions is such that an intervention newly approved for funding by a PSI will replace a legacy intervention which was approved by the PSI in a previous funding cycle. While it has not yet been possible to extend the current model to represent this situation, such an extension would provide a more accurate understanding of the cumulative benefits which are gained from a PSI through time.

Another factor which is not incorporated into the current model is the potential influence which a PSI can have on upstream negotiation with a manufacturer/producer of a new intervention. A threshold-based approach is completely transparent, and therefore presents manufacturers with a specific performance target (in terms of both costs and health benefits) which they must achieve in order to receive funding approval for their intervention. This scenario gives the PSI a clear negotiating position with the manufacturers, and puts the onus on the manufacturers to construct a more attractive product offering if they cannot satisfy the threshold (for example through reducing profit margins, or providing societal benefits through alternative mechanisms).

The methodology and analysis presented here only represent an overview of the contribution simulation models could make to support funding decisions related to both new and established PSIs. Such models could be used to understand the impact of a wide variety of funding choices, providing quantified measures of the effectiveness of these. The two indicative data-sets considered for analysis demonstrate the potential variations in performance which could be realised for different inputs to the simulation model. This highlights the importance of carefully parameterising the model inputs, and for appropriate selection and interpretation of the distributional models and dependence structure of the data.

Of course, many of the benefits of implementing PSIs cannot be captured in a quantitative simulation such as this, and in a sister study we focus on the qualitative benefits [19]. For example, having a rigorous assessment process ensures that clinical guidelines can be written in a clear and practical way, and can easily be implemented by clinicians. The information collected by the PSI also improves the general understanding of intervention outcomes, making it easier to conduct equity analysis. Additionally, the transparency associated with taking a formal evaluation approach also lessens the scope for corruption in decision making, where funding decisions are not being made on a fair and equitable basis. The random intervention selection that is modelled through our PSI-absent scenarios (where interventions are not funded on the basis of costs or benefits) may be broadly reflective of an unfair decision making process, although formally modelling this behaviour would be difficult to parameterise. In either case, taking a qualitative perspective on the impact of unfair decisions may provide more useful insights. A potential limitation of purely CER-based decisions is that these may deteriorate health equity across population groups. To mitigate this, a PSI may include a topic selection process prior to cost-efficiency assessments (see for example Youngkong et al. [29] for the case of Thailand). Modelling topic selection could readily be incorporated into the Section 3 model, by including an extra dimension to the intervention specification and decision criteria.

## Conclusion

Simulation methods are underused in quantifying the economic and health impact of priority setting institutionalisation. This study represents a first step along the road that would help convince governments to invest in PSI (especially in LMICs, as HIC governments have widely recognised the importance of PSI), and to show the potential of simulation methods to answer policy relevant questions. There are many ways to refine the simulation results but our overall finding is consistent with other literature: the benefits of explicit priority setting are substantial in both resource-limited and resource-rich settings. To help others build on the work, we provide a useable spreadsheet tool and make it publicly available [20] so we encourage readers to download the tool and explore the simulations for themselves.

## Figures and Tables

**Fig. 7:**
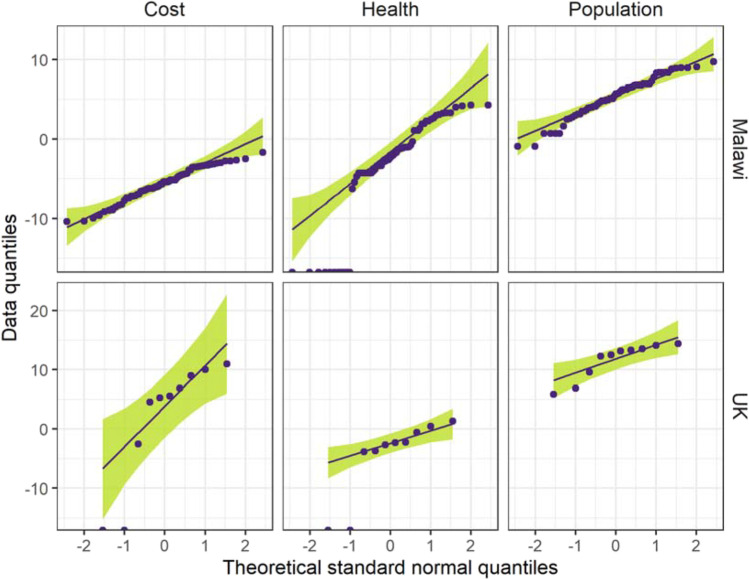
Comparison of the (log-transformed) indicative Malawi and UK data-sets with normally distributed data. For each country, the plots show the indicative data on the three aspects of an intervention: the incremental costs of administering each intervention per treated case (Cost), the incremental health benefits returned per case treated with the intervention (Health), and the number of cases treated by each intervention (Population). The indicative Malawi and UK data-sets are taken from Ochalek et al. [23] and Guthrie et al. [10], respectively, and are presented in Appendix 1

**Fig. 8:**
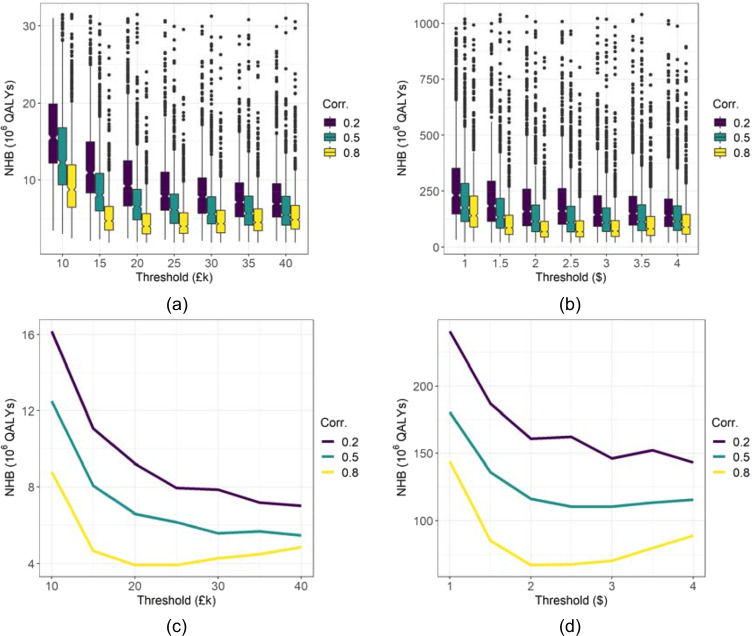
${NHB({I}_{TH},I}_{CF},t)$ as defined in Eq. (11), with portfolio ${I}_{TH}$ determined using threshold funding decision rule (5) and portfolio ${I}_{CF}$ determined using counterfactual rule (3), recorded as threshold $t$ varies in (5), for three levels of correlation ${\rho }_{CQ}$. (**a**) Indicative UK data: full distributions, (**b**) Indicative Malawi data: full distributions, (**c**) Indicative UK data: median values, (**d**) Indicative Malawi data: median values

**Fig. 9:**
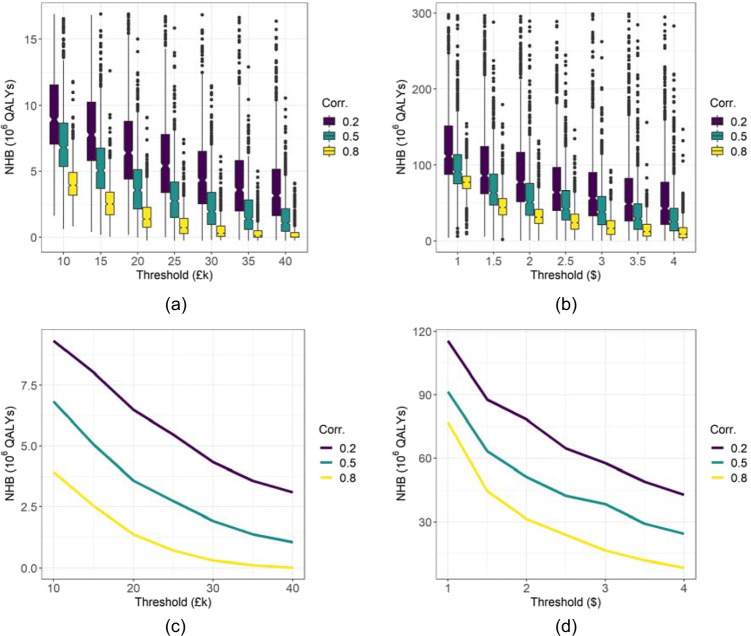
${NHB({I}_{TH},I}_{CF},t)$ as defined in Eq. (11), with portfolio ${I}_{TH}$ determined using threshold funding decision rule (6) and portfolio ${I}_{CF}$ determined using counterfactual rule (4), recorded as threshold $t$ varies in (6), for three levels of correlation ${\rho }_{CQ}$. (**a**) Indicative UK data: full distributions, (**b**) Indicative Malawi data: full distributions, (**c**) Indicative UK data: median values, (**d**) Indicative Malawi data: median values

**Fig. 10:**
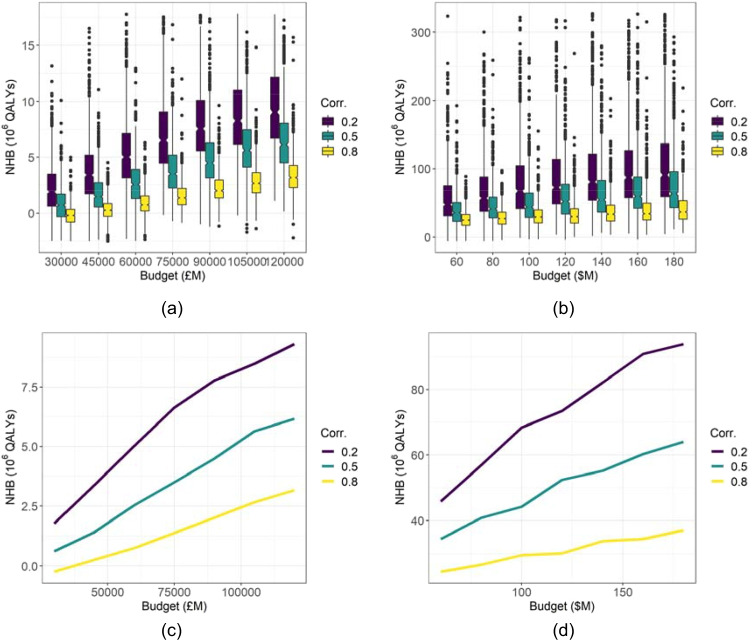
${NHB({I}_{TH},I}_{CF},t)$ as defined in Eq. (11), with portfolio ${I}_{TH}$ determined using threshold funding decision rule (6) and portfolio ${I}_{CF}$ determined using counterfactual rule (4), recorded as budget limit $l$ varies in (6), for three levels of correlation ${\rho }_{CQ}$. (**a**) Indicative UK data: full distributions, (**b**) Indicative Malawi data: full distributions, (**c**) Indicative UK data: median values, (**d**) Indicative Malawi data: median values

**Fig. 11:**
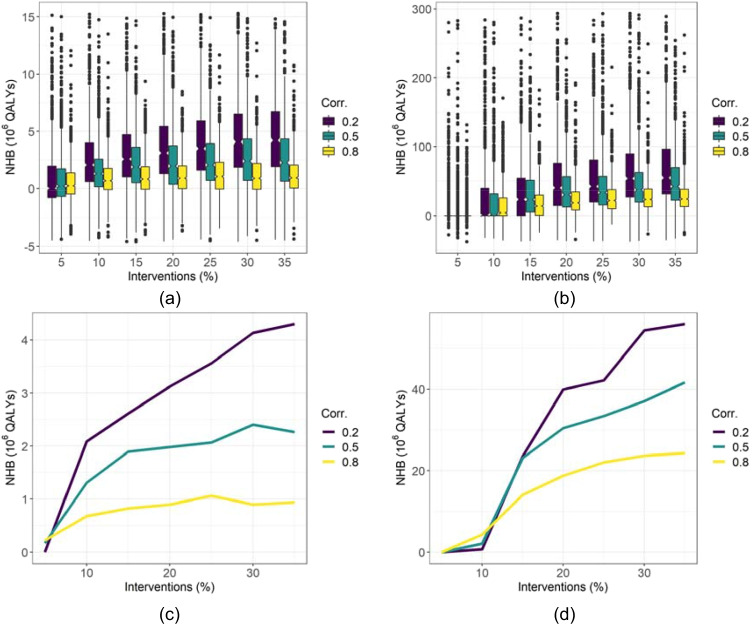
${NHB({I}_{TH},I}_{CF},t)$ as defined in Eq. (11), with portfolio ${I}_{TH}$ determined using threshold funding decision rule (7) and portfolio ${I}_{CF}$ determined using counterfactual rule (4), recorded as percentage ${p}_{a}$ varies in (7), for three levels of correlation ${\rho }_{CQ}$. (**a**) Indicative UK data: full distributions, (**b**) Indicative Malawi data: full distributions, (**c**) Indicative UK data: median values, (**d**) Indicative Malawi data: median values

**Fig. 12:**
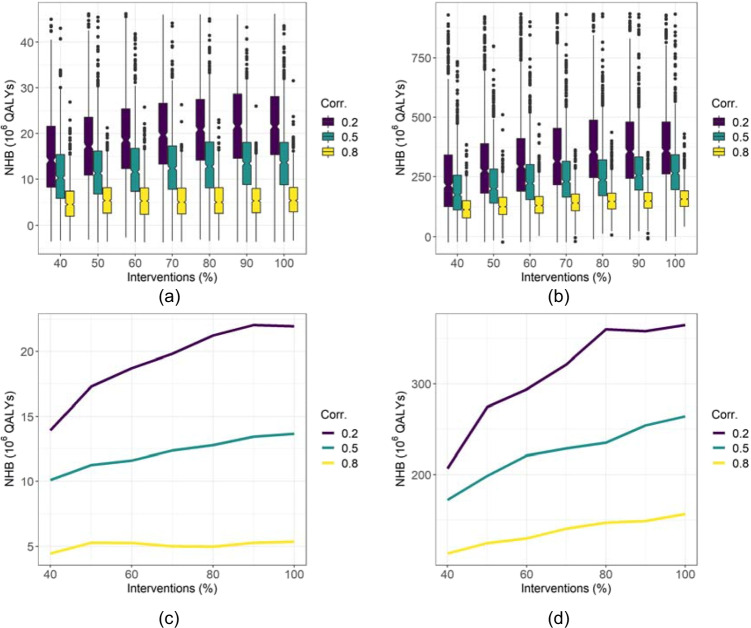
${NHB({I}_{TH},I}_{CF},t)$ as defined in Eq. (11), with portfolio ${I}_{TH}$ determined using threshold funding decision rule (8) and portfolio ${I}_{CF}$ determined using counterfactual rule (4), recorded as percentage ${p}_{Y}$ varies in (8), for three levels of correlation ${\rho }_{CQ}$. (**a**) Indicative UK data: full distributions, (**b**) Indicative Malawi data: full distributions, (**c**) Indicative UK data: median values, (**d**) Indicative Malawi data: median values

**Fig. 13:**
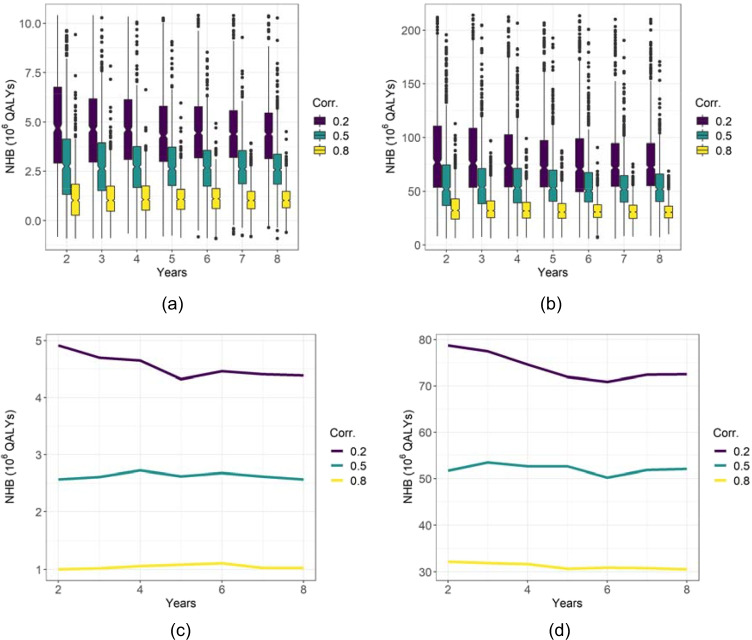
${NHB({I}_{TH},I}_{CF},t)$ as defined in Eq. (11), with portfolio ${I}_{TH}$ determined using threshold funding decision rule (8) and portfolio ${I}_{CF}$ determined using counterfactual rule (4), recorded as the number of funding cycles $Y$ varies in (8), for three levels of correlation ${\rho }_{CQ}$. (**a**) Indicative UK data: full distributions, (**b**) Indicative Malawi data: full distributions, (**c**) Indicative UK data: median values, (**d**) Indicative Malawi data: median values

## Data Availability

The data used for the analysis is provided in the Appendices.

## Appendix 1

The indicative data-set for UK health interventions is taken from Guthrie et al. [10], and is shown in Table 9.

**Table 9:** Table of indicative heath interventions considered for funding in the UK, extracted from Guthrie et al. [10]

${{i}}$	Study Authors	QALY increment $({{{Q}}}_{{{i}}})$	Cost increment (2012 prices) $({{{C}}}_{{{i}}})$	Number of cases in the UK $({{{P}}}_{{{i}}})$
1	Peek et al. (2010)	3.66	59,415.7	350
2	Carroll et al. (2011)	0.58	7952	14,479
3	Vickers et al. (2004)	0.021	252.4	723,236
4	Gilbert et al. (2004)	0.07	92.85	260,000
5	McCarthy et al. (2004)	0	-8	1,322,709
6	Cochrane et al. (2005)	0.024	0.08	1,763,613
7	Lamb et al. (2010)	0.099	196	598,000
8	Pandor et al. (2013)	0.1038	992	504,689
9	Orlando et al. (2013)	1.6239	22,528	947
10	Kitchener et al. (2009)	0	-14	214,138

The authors state the total number of health interventions considered for funding over a 10 year period as 740, and from this the average number per year (*N*) is assumed to be 74. The authors also discuss the widely-used UK threshold of the cost-effectiveness ratio for funding approval $(t)$ as £20,000. The remaining decision parameters for the Case (i)—Case (iv) investigations are selected such that the extensions to the decision rules have an observable impact on the simulation outputs. To define these decision parameters, an initial run of the Case (i) PSI-active scenario with the middle correlation level $({\rho }_{CQ}=0.5)$ is simulated, and is used as a benchmark investigation for setting the decision parameters in other investigations. For the Case (i) PSI-absent scenario, the number of funded interventions is set to the average number of interventions funded under the benchmark investigation (*n* = 48). For the Case (ii) investigations, the available budget limit (*l*) is chosen such that fewer interventions will be funded (that is, ensuring that the budget limit is an active funding constraint). For both Case (ii) funding scenarios, the available budget limit is set to *l*=£75,000M, approximately 50% of the average total cost of funded interventions per simulation for the benchmark investigation. For the Case (iii) PSI-active scenario investigation, the annual proportion of highest budget-impacting interventions that a CER assessment is applied to is set to ${p}_{a}=20\%$ – this value was chosen to impose a further reduction on the number of interventions that would be funded under the Case (ii) investigation. For the Case (iv) PSI-active scenario investigation, the number of years *Y* is set to 5 such that in year 5 ${p}_{Y}=100\%$, and all interventions are subject to the CER assessment.

The indicative data-set for Malawi health interventions is taken from Ochalek et al. [23], and is shown in Table

**Table 10:** Table of indicative heath interventions considered for funding in Malawi, extracted from Ochalek et al. [23]

$$i$$	Intervention	Total DALYS averted $${(QT}_{i})$$	Total cost ($1000 s) $${(CT}_{i})$$	Cases per annum (1000 s) $${(P}_{i})$$
1	Male circumcision	39,634	146,730	4073
2	Management of obstructed labour	2497	1100	92
3	Isoniazid preventive therapy for HIV + no TB	1118	80	55
4	First-line treatment for new TB cases for adults	1045	178	14
5	First-line treatment for new TB cases for children	888	117	12
6	Management of pre-eclampsia (magnesium sulfate)	535	45	20
7	Clean practices and immediate essential newborn care (home)	237	416	671
8	Households owning at least one ITN/LLIN	228	13,737	6752
9	Caesarean section	327	672	34
10	Mass media	150	7609	16,879
11	Labour and delivery management	170	1281	918
12	PMTCT of HIV	157	600	53
13	First-line treatment for retreatment TB cases for adults	131	100	2
14	Caesarean section (with complication)	137	172	5
15	First-line treatment for retreatment TB cases for children	111	66	2
16	Malaria treatment: first trimester— uncomplicated	109	1025	305
17	Malaria treatment: Second trimester—uncomplicated	109	235	305
18	Voluntary counselling and testing	167	36,309	8031
19	Tetanus toxoid (pregnant women)	104	115	918
20	Measles vaccine	107	528	651
21	Rotavirus vaccine	88	3097	651
22	Antenatal care (four visits)	90	11,230	918
23	Malaria treatment: uncomplicated (adult, < 36 kg)	59	3463	4372
24	Malaria treatment: uncomplicated (adult, > 36 kg)	59	4267	4372
25	Malaria treatment: uncomplicated—second line (adult, > 36 kg)	59	1186	4372
26	Malaria treatment: uncomplicated—second line (adult, < 36 kg)	59	593	4372
27	Vaginal delivery, skilled attendance	67	5181	918
28	Isoniazid preventive therapy for children in contact with patients with TB	45	7	2
29	Interventions focused on men who have sex with men	232	1256	34
30	Pregnant women sleeping under an ITN	50	2990	1469
31	Newborn sepsis—full supportive care	60	417	81
32	Management of severe malnutrition (children)	199	2437	51
33	Vitamin A supplementation in pregnant women	33	125	124
34	Antenatal corticosteroids for preterm labour	47	406	165
35	Interventions focused on female sex workers	161	655	23
36	Cotrimoxazole for children	0	220	127
37	Malaria treatment: uncomplicated (children, < 15 kg)	14	4576	1042
38	Malaria treatment: uncomplicated (children, > 15 kg)	14	4768	1042
39	Malaria treatment: uncomplicated—second line (children, < 15 kg)	14	35	1042
40	Malaria treatment: uncomplicated—second line (children, > 15 kg)	14	71	1042
41	Under five children who slept under ITN/LLIN	17	1006	494
42	Schistosomiasis mass drug administration	24	77	389
43	Antibiotics for pPRoM	30	39	64
44	Blood safety	12	1626	40
45	Vaginal delivery, with complication	10	804	138
46	Maternal sepsis case management	20	2731	64
47	Malaria treatment: pregnant Women —complicated	6	140	16
48	Case management of MDR TB cases	5	12	0.4
49	GIT tract cancer	0	3	0.4
50	Cervical cancer (first line)	0	162	2
51	Ischaemic heart disease	0	4	128
52	IPT of malaria (pregnant women)	0	35	735
53	Diabetes, type I	0	4304	23
54	High cholesterol	1	6703	223
55	Basic psychosocial support, advice and follow-up, plus antiepileptic medication	1	1266	506
56	Treatment of depression	0	332	169
57	Diabetes, Type II	0	4211	138
58	Treatment of acute psychotic disorders	0	958	169
59	Treatment of bipolar disorder	0	10,362	523
60	Treatment of schizophrenia	0	13,413	2363
61	Hypertension	44	1338	846
62	Zinc (diarrhoea treatment)	244	1788	7455
63	ORS	147	937	8662
64	Condoms	482	22,883	8031
65	ART for men	1005	21,159	332
66	ART for women	1541	32,440	509
67	Paediatric ART	1556	7657	107

^10^. Data could not be obtained on the number of interventions considered for funding per cycle $$(N)$$, and for the purposes of modelling this was set to 40 per year to represent a smaller set of interventions than for the UK data. Ochalek et al. [23] state that in Malawi the accepted threshold of the cost-effectiveness ratio for funding approval is at least $61. Using this threshold, however, would result in the vast majority of interventions being funded. In order to investigate the differences which could arise between implementing counterfactual and threshold decision rules, the cost-effectiveness ratio threshold for funding approval $$(t)$$ was substantially reduced, to $2 which is the average cost-effectiveness ratio of the interventions listed in Table 10. As for the UK data, the remaining decision parameters are selected such that these have an observable impact on the simulation outputs, and an initial simulation run of the Case (i) PSI-active scenario with the middle correlation level ($${\rho }_{CQ}=0.5$$) is used as a benchmark investigation for setting these decision parameters. For the Case (i) PSI-absent scenario, the number of funded interventions is again set to the average number of interventions funded under the benchmark investigation ($$n=14$$). For the Case (ii) investigations, the available budget limit is again set to approximately 50% of the average total cost of funded interventions per simulation for the benchmark investigation ($$l=\$150M$$). The decision parameters for the Case (iii) and Case (iv) PSI-active scenario investigations are set to the values for the UK data.

## Appendix 2

Consider two independent standard normal variables, ${Z}_{1}\sim N({0,1})$ and $\hat{Z}\sim N({0,1})$. An additional standard normal variable ${Z}_{2}\sim N({0,1})$ is then defined such that

$${Z}_{2}={\rho }_{X}{Z}_{1}+\sqrt{(1-{{\rho }_{X}}^{2})}\hat{Z}$$

The variable ${Z}_{2}$ is therefore a linear combination of the independent variables ${Z}_{1}$ and $\hat{Z}$, such that the value of ${Z}_{2}$ consists of a weighting ${\rho }_{X}$ of ${Z}_{1}$. That is, a proportion ${\rho }_{X}$ of the value of ${Z}_{2}$ is associated with the value of ${Z}_{1}$ and the remainder is independent of this, driven by the value of $\hat{Z}.$ This definition of ${Z}_{2}$ therefore imposes a linear correlation of ${\rho }_{X}$ between ${Z}_{1}$ and ${Z}_{2}$. These standard normal variables are translated into correlated normal variables with specified distributions by defining

$${\displaystyle \begin{array}{c}{X}_1={\mu}_{X_1}+{Z}_1{\sigma}_{X_1}\\ {}{X}_2={\mu}_{X_2}+{Z}_2{\sigma}_{X_2},\end{array}}$$

such that ${X}_{i}\sim N\left({\mu }_{{X}_{i}},{{\sigma }_{{X}_{i}}}^{2}\right)$ for $i={1,2}$. That is, ${X}_{1}$ and ${X}_{2}$ are normally distributed with mean ${\mu }_{{X}_{1}}$ and ${\mu }_{{X}_{2}}$, respectively, and standard deviation ${\sigma }_{{X}_{1}}$ and ${\sigma }_{{X}_{2}}$, respectively. Note that the properties of the normal distribution are such that the level of correlation between ${X}_{1}$ and ${X}_{2}$ is also ${\rho }_{X}$.

The correlated normal variables are transformed into correlated log-normal variables by defining

$${\displaystyle \begin{array}{c}{Y}_1=\exp \left({X}_1\right)\\ {}{Y}_2=\exp \left({X}_2\right).\end{array}}$$

Expanding this gives

$${Y}_{1}=\exp \left({{\mu }_{{X}_{1}}+Z}_{1}{\sigma }_{{X}_{1}}\right)$$

and

$${Y}_{2}=\exp \left({\mu }_{{X}_{2}}+{\sigma }_{{X}_{2}}\left({\rho }_{X}{Z}_{1}+\sqrt{(1-{{\rho }_{X}}^{2})}\hat{Z}\right)\right).$$

The correlation between any two random variables ${Y}_{1}$ and ${Y}_{2}$ is defined as

$${\rho }_{Y}=\frac{\text{Cov}({Y}_{1},{Y}_{2})}{{\sigma }_{{Y}_{1}}{\sigma }_{{Y}_{2}}},$$

where $\text{Cov}({Y}_{1},{Y}_{2})$ denotes the covariance between ${Y}_{1}$ and ${Y}_{2}$. This can be written in terms of the arithmetic moments of the variables as

$${\rho }_{Y}=\frac{\mathrm{E}\left[{Y}_{1}{Y}_{2}\right]-\mathrm{E}\left[{Y}_{1}\right]\mathrm{E}\left[{Y}_{2}\right] }{\mathrm{SD}\left[{Y}_{1}\right]\mathrm{SD}\left[{Y}_{2}\right]},$$

where $\mathrm{E}\left[Y\right]$ and $\mathrm{SD}\left[Y\right]$ respectively represent the arithmetic mean and standard deviation of a variable $\mathrm{Y}$. As ${Y}_{1}$ and ${Y}_{2}$ are log-normally distributed, then these arithmetic moments are well defined [12], and the correlation can be written as

$${\rho }_{Y}=\frac{\exp \left({\mu }_{{X}_{1}}+{\mu }_{{X}_{2}}+0.5({{\sigma }_{{X}_{1}}}^{2}+{{\sigma }_{{X}_{2}}}^{2}+2{\sigma }_{{X}_{1}}{\sigma }_{{X}_{2}}{\rho }_{X})\right)-\exp \left({\mu }_{{X}_{1}}+0.5{{\sigma }_{{X}_{1}}}^{2}\right)\exp \left({\mu }_{{X}_{2}}+0.5{{\sigma }_{{X}_{2}}}^{2}\right) }{\exp \left({\mu }_{{X}_{1}}+0.5{{\sigma }_{{X}_{1}}}^{2}\right)\sqrt{\left(\exp \left({{\sigma }_{{X}_{1}}}^{2}\right)-1\right)}\mathrm{ exp}\left({\mu }_{{X}_{2}}+0.5{{\sigma }_{{X}_{2}}}^{2}\right)\sqrt{\left(\exp \left({{\sigma }_{{X}_{2}}}^{2}\right)-1\right)}}.$$

This reduces to

$${\rho }_{Y}=\frac{\exp \left({\sigma }_{{X}_{1}}{\sigma }_{{X}_{2}}{\rho }_{X}\right)-1}{\sqrt{\left(\exp \left({{\sigma }_{{X}_{1}}}^{2}\right)-1\right)} \sqrt{\left(\exp \left({{\sigma }_{{X}_{2}}}^{2}\right)-1\right)}}.$$

Suppose that two log-normal variables are defined as ${Y}_{1}\sim LogN({\mu }_{{Y}_{1}},{{\sigma }_{{Y}_{1}}}^{2})$ and ${Y}_{2}\sim LogN({\mu }_{{Y}_{2}},{{\sigma }_{{Y}_{2}}}^{2})$, such that ${Y}_{1}$ and ${Y}_{2}$ have means ${\mu }_{{Y}_{1}}$ and ${\mu }_{{Y}_{2}}$, respectively, and standard deviations ${\sigma }_{{Y}_{1}}$ and ${\sigma }_{{Y}_{2}}$, respectively. Suppose further that the level of correlation between these variables is specified as ${\rho }_{Y}$. Then, defining

$${{\sigma }_{{X}_{i}}}^{2}=\text{ln}\left(1 + \frac{{{\sigma }_{{Y}_{i}}}^{2}}{{{\mu }_{{Y}_{i}}}^{2}}\right)\text{ and }\:{\mu }_{{X}_{i}}=\text{ln}\left({\mu }_{{Y}_{i}} \right)-0.5{{\sigma }_{{X}_{i}}}^{2}$$

for $$i={1,2}$$ and

$${\rho }_{X}=\frac{\text{ln}\left({\rho }_{Y}\sqrt{\left(\exp \left({{\sigma }_{{X}_{1}}}^{2}\right)-1\right)\left(\exp \left({{\sigma }_{{X}_{2}}}^{2}\right)-1\right)}+1\right)}{{{\sigma }_{{X}_{1}}}^{2}{{\sigma }_{{X}_{2}}}^{2}},$$

the above process can be implemented to generate random samples from the distributions of ${Y}_{1}$ and ${Y}_{2}$ which will have the desired correlation of ${\rho }_{Y}$. This process was used to generate the samples of implementation cost and health benefit for a set of interventions, which have a specified level of correlation.

## Appendix 3

A preliminary analysis of the indicative data-sets demonstrates that, per intervention, the incremental costs of administering each intervention per treated case, the incremental health benefits returned per case treated with the intervention, and the number of cases treated by each intervention, each have a positive-skewed distribution when analysed across the interventions in each country. Each of these characteristics are also predominantly positive, and the log-normal is therefore a natural choice to model the distributions in each case. Furthermore, the log-normal distribution can be fully parameterised with only the mean and standard deviation of each characteristic, which are comparatively straightforward and intuitive properties to extract from data or to elicit. Figure 7 shows Q-Q plots (see for example Thode [27]) comparing the (log-transformed) indicative data-sets with the theoretical quantile values expected for normally distributed data. Overall, the data points show a reasonable fit to the line of perfect fit, particularly given the relatively small sample size for the UK data. We therefore assume that each of these intervention characteristics is log-normally distributed, and specify these as $P\sim LogN({\mu }_{P},{{\sigma }_{P}}^{2})$, $C\sim LogN({\mu }_{C},{{\sigma }_{C}}^{2})$ and $Q\sim LogN({\mu }_{Q},{{\sigma }_{Q}}^{2})$, where $\mu$ and $\sigma$ represent the mean and standard deviation of each distribution, and the subscripts correspond to the respective random variables. These stochastic model parameters are summarised in Table 3, and the distribution parameters and other inputs are defined in Table 2.

A limitation with this assumption is demonstrated by the small number of points shown along the bottom edge of the plots, which represent data values less than or equal to zero, that cannot be represented by the strictly-positive log-normal distribution. However, this limitation only applies to a relatively small portion of the indicative data – comprising 20% of the UK “Cost” data, 20% of the UK “Health” data, and 16% of the Malawi “Health” data. Additionally, these data values are incorporated into the mean and standard deviation calculations for each data-set (see Table 2), and therefore still have an influence on the shape of the distribution. The impact of this limitation is that the samples of these stochastic model parameters (see Table 3) can be expected to be marginally greater than is true to the distribution of the data, and the overall effect on the model outputs is expected to be minimal. A further limitation with this assumption is that the log-normal distribution will generate continuous samples for the discrete population impact per intervention. The population impact in both indicative data-sets, however, is typically in the thousands. Additionally, the role of the population impact is to scale the intervention’s health benefits and implementation costs to a population level, and so the impact of non-integer samples for the population impact can be expected to be negligible.

## Appendix 4

Given the indicative nature of the UK and Malawi datasets, and the differences observed between the analysis of the decision rules as applied to these, it is of interest to explore the impact of varying the decision rule parameters. Furthermore, it is of interest to investigate these impacts as the level of correlation between the intervention costs and benefits varies. Each set of figures below represents an investigation varying a single decision parameter, and repeats this for three levels of correlation ${\rho }_{CQ}$ between intervention costs and health benefits, and for both the indicative UK and Malawi data-sets. The set of values explored for each decision parameter are provided in Table

**Table 11:** Input parameters used for the Appendix 4 investigations. For reference, the values marked in bold represent the parameter value used in Section 4

Investigation	Input parameter (symbol)	Country data-set	Set of investigated input parameter values	Units
Case (i)	CER threshold required for funding $(t)$	UK	$$\left\{\begin{array}{c}{10,15},20,25,\\ {30,35,40}\end{array}\right\}$$	£k
		Malawi	$\{{1.0,1.5},2.0,2.5,$ ${3.0,3.5,4.0}\}$	${\$}$
Case (ii)	CER threshold required for funding $(t)$	UK	$$\left\{\begin{array}{c}{10,15},20,25,\\ {30,35,40}\end{array}\right\}$$	£k
		Malawi	$\{{1.0,1.5},2.0,2.5,$ ${3.0,3.5,4.0}\}$	${\$}$
	Budget limit for funding interventions $(l)$	UK	$$\left\{\begin{array}{c}{30,45,60,7}5,\\ {90,105,120}\end{array}\right\}$$	£Mx10^3^
		Malawi	$\{{60,80,100},120,$ ${140,160,180}\}$	${\$}\mathrm{M}$
Case (iii)	Percentage of high-value interventions assessed using the threshold rule $(p)$	Both	$\{{5,10,15},20,$ ${25,30,35}\}$	%
Case (iv)	Target percentage of interventions assessed by the PSI in the final year of phased increases $(p)$	Both	$\{{40,50,60,70},$ ${80,90},100\}$	%
	Total number of years of phased increases to the PSI capability $(Y)$	Both	$\{{2,3},4,5,{6,7},8\}$	Years

11. The candidate set ${I}_{A}$ consists of 100 randomly sampled interventions in each simulation. For each combination of decision parameter and correlation level 1000 simulations are run, and the distribution of selected outputs across all simulations are summarised with box-plots. The highest and lowest 1% of values from each collection of distributions are removed from the plots to provide a clearer understanding of the mass of the distributions. The NHB is used as the output metric to compare the decision rules.

### Case (i): Sensitivity analysis for the threshold rule

In Fig. 8, decision rules (3) and (5) are compared as the CER threshold *t*, below which interventions are funded, is varied. The overall trend across both data-sets is that initially as $$t$$ increases the NHB decreases (doing so more rapidly for higher $${\rho }_{CQ}$$) but eventually the NHB becomes relatively constant (again, this stable NHB occurs more rapidly for higher $${\rho }_{CQ}$$). The range of the NHB distributions, interquartile ranges (IQRs), and the confidence interval around the median follow the same trend, in each case decreasing as *t* and $${\rho }_{CQ}$$ increase. Prior to stabilising, the confidence intervals around the medians indicate that these are significantly different. Increasing $$t$$ will result in additional interventions being funded, and these additional interventions will represent a poorer cost-effectiveness return, particularly with lower $${\rho }_{CQ}$$ as higher costs will be less likey to deliver higher benefits. A noticeable feature of Fig. 8 is that, with a high correlation level, the NHB displays a minimum value (for $$t$$ approximately equal to £20k for the indicative UK data and $2 for the indicative Malawi data).

### Case (ii): Sensitivity analysis for the threshold rule with budget constraint

Figure 9 compares decision rules (4) and (6) as threshold *t* varies, and the same decision rules are compared in Fig. 10 as the available funding budget limit $l$ varies. The trends observed in Fig. 9 are broadly comparable to those observed in Fig. 8, although with a budget limit in place the realized values of NHB are noticeably reduced in Fig. 9 (as some interventions will go unfunded). The trends observed in Fig. 10 are to be expected, with incremental changes to the budget limit resulting in incremental changes to the NHB value; if the budget was increased further the gains in NHB would be expected to plateau as the value tends towards the non-budgeted case (decision rule (5)).

### Case (iii): Sensitivity analysis for the threshold rule with limited analysis capacity

In Fig. 11, decision rules (4) and (7) are compared as the percentage ${p}_{a}$ of high-value interventions assessed using the threshold rule increases. The overall trend is that as ${p}_{a}$ increases the NHB increases, and with more interventions assessed in terms of a CER a natural conclusion is that the NHB would increase. Increasing ${p}_{a}$ has a diminishing impact on the NHB, for higher levels of correlation, the gains returned from increasing ${p}_{a}$ are negligible.

### Case (iv): Sensitivity analysis for the threshold rule with phased run-in

Figures 12 and 13 compare decision rules (4) and (8); Fig. 12 demonstrating the impact of varying the final target percentage ${p}_{Y}$, and Fig. 13 demonstrating the impact of varying the number of funding cycles *Y*. In Fig. 12 the two data-sets are broadly comparable, however, the level of correlation ${\rho }_{CQ}$ is again shown to have a substantial impact on the variation of the NHB value. Similar to Fig. 11, there is a diminishing impact from increasing ${p}_{Y}$, and for higher correlation levels the final percentage ${p}_{Y}$ is shown to have a relatively minor impact on NHB. Figure 13 indicates that there is little observable impact on the realized NHB (calculated as an average per funding cycle) when the number of funding cycles used to reach a steady operating state are varied. Comparison of the confidence intervals around the median for each box-plot reveals that there is much overlap between these, which indicates that there is limited evidence of statistical differences between the medians. What is evident, however, is that the range and IQR of the NHB distributions reduces as the number of funding cycles increases.

## Code availability

The interactive simulation model is publicly accessible here (see Morton and Barlow [20])
